# Effect of Welding Parameters on Mechanical Properties and Microstructure of Friction Stir Welded AA7075-T651 Aluminum Alloy Butt Joints

**DOI:** 10.3390/ma15175950

**Published:** 2022-08-28

**Authors:** Robert Kosturek, Janusz Torzewski, Marcin Wachowski, Lucjan Śnieżek

**Affiliations:** Faculty of Mechanical Engineering, Military University of Technology, 2 Gen. S. Kaliskiego Str., 00-908 Warsaw, Poland

**Keywords:** friction stir welding, aluminum, welding parameters, mechanical properties, microstructure, fracture

## Abstract

The aim of this study was to examine the mechanical properties of 5-mm-thick AA7075-T651 alloy using three different welding velocities, 50, 75 and 100 mm/min, and four various sets of tool rotation speeds: 400, 600, 800 and 1000 rpm. All obtained joints were defect-free. In all cases, the values of UTS exceeded 400 MPa, corresponding to 68.5% minimum joint efficiency. The highest value of 447.7 MPa (76.7% joint efficiency) was reported for the joint produced via 400 rpm tool rotation speed and 100 mm/min welding velocity. The SZ microstructure of the strongest joint was characterized by a 5.2 ± 1.7 μm grain size and microhardness of approximately 145 HV0.1. The TMAZ/HAZ interface was identified as the low-hardness zone (105–115 HV0.1, depending on parameters), where the failure of the tensile samples takes place. The fracture mechanism is dominated by a transgranular ductile rupture with microvoid coalescence.

## 1. Introduction

Among the aluminum alloys used in the aircraft and military industries, AA7075-T651 alloy has one of the highest strength parameters [1]. The alloy is used as a construction material for stringers, wing ribs, missile parts, etc., due to its very high load carrier capacity with relatively low density (2.81 g/cm^3^) [2,3]. In terms of joining components made of 7075, the most common method is riveting [4]. It is partly dictated by the very poor weldability of this alloy by traditional welding processes [5,6]. In recent years, friction stir welding (FSW) has been introduced as a very efficient technology in joining aluminum alloys in solid state by the local stirring of a plasticized material from both elements to be welded [7,8,9]. The overall performance of aluminum alloy FSW joints is very good, but when it comes to the precipitation-hardened alloys (2XXX and 7XXX series), a reduction in mechanical properties is inevitable [8,10,11,12]. The reason is directly concerned with unfavorable evolutions of the strengthening phase undergoing during a thermal cycle of the welding process [13]. Eventually, the high temperature leads to losses in the strengthening phases and the strength of the joint is lower than that of the base material. As for 2XXX alloys, strengthened by GP zones or the θ′ phase, the joint efficiency that can be achieved is even 80–90%; in the case of 7XXX alloys, including 7075-T651, strengthened by the η′ phase, the obtained joint efficiency rarely exceeds 75% [8,14,15].

Many research papers about the joining of 7075-T651 via FSW can be found in the literature [16,17,18,19,20,21,22]. Dimopoulos et al. investigated 2-mm-thick 7075-T651 jointed via FSW with various sets of welding parameters and tools and reported at least a 33.75% decrease in the ultimate tensile strength (UTS) in relation to the base material [16]. Moreover, Yeni et al. focused on increasing 7075 FSW weld strength by post-weld aging and, in the best variant, obtained a value of 63% joint efficiency [17]. Fuller et al. researched changes in the microstructure and mechanical properties of 7075 naturally aged FSW joints in the form of 6.35- and 3.175-mm-thick plates and stated that the thinner plate exhibited increased tensile strength due to a faster workpiece cooling rate [18]. On the other hand, Fratini et al. performed a study concerning material flow in AA7075-T6 FSW butt joints and reported that conical pins determine a more effective material flow in FSW, allowing the avoidance of the insurgence of flow defects [19]. Moreover, Iwaszko et al. examined the air-cooled friction stir processing of AA7075 and concluded that intensifying the cooling process leads to greater grain refinement and a more homogeneous microstructure, including the redistribution of intermetallic phase precipitates [20]. Furthermore, Zhang et al. studied high-speed FSW of AA7075-T6 (up to 3 m/min) and stated that welds produced with a higher welding speed generally have a smaller softened region and lower hardness decrease in the heat-affected zone (HAZ) [21]. Moreover, Li et al. investigated stationary shoulder FSW (SSFSW) of AA7075-T651 and, in the best variant, obtained 69% joint efficiency [22]. Additionally, Sun et al. performed a tensile and fatigue analysis of AA7075 FSW joints and reported the largest deformation in the thermo-mechanically affected zone (TMAZ), where the low-hardness zone (LHZ) was localized [23].

Generally, both increasing welding velocity and additional cooling give positive effects in terms of microstructure but, on the other hand, also increase residual stresses in welded elements [20,21,24]. Analyzing the used welding velocities, in many cases, the lowest value is 80 mm/min and the tool rotation speed exceeds 800 rpm [16,24]. Moreover, many investigations focus mainly on microstructural aspects (the evolutions of the strengthening phase, participation of grain boundaries in each welded zone), simultaneously failing to provide the mechanical properties data in a broad range of welding parameters. An additional factor that should be considered is the fact that the obtained properties depend on the thickness of workpieces to be welded [7,18]. Much attention is paid to 2-mm-thick high-strength aluminum alloys due to their aircraft sheeting applications, which generally give relatively high mechanical properties in terms of the FSW joints [7,16,21,25]. Although, for thicker workpieces, plenty of research can be found in the literature, it is often concerned with 6.35-mm-thick elements [15,18,26]. When it comes to investigations on 5-mm-thick AA7075-T651, a relatively low number of works are available. Data provided by Liu for 5-mm-thick AA7075-T651 cover the SSFSW technique, which is characterized by differences in heat distribution and plastic flow and cannot be directly extrapolated to the conventional FSW process [22,24]. In this field, Rajakumar et al. contributed significantly, performing a study on the FSW of 5-mm-thick AA7075-T6 alloy in the following range of welding parameters: 900–1800 rpm tool rotation speed and 20–100 mm/min tool traverse speed [27]. In the best set of welding parameters (1400 rpm, 60 mm/min), the authors achieved 77% joint efficiency, which is a very good result [27]. Nevertheless, the UTS of their base material (AA7075-T6) was only 485 MPa, which is almost 100 MPa less than in the case of AA7075-T651 alloy [22,27]. For this reason, the mechanical properties of 5-mm-thick AA7075-T651 FSW joints examined in a broad range of welding parameters still constitute a scientific gap in the current state of the art.

In this investigation, we aim to examine the mechanical properties of 5-mm-thick AA7075-T651 alloy using three different welding velocities, 50, 75 and 100 mm/min, and four various sets of tool rotation speeds: 400, 600, 800 and 1000 rpm. The mechanical properties are established in tensile tests, and, based on the obtained results, a set of samples is selected for macro- and microstructural analysis. The joints that provide the highest and the lowest values of the UTS are subjected to microhardness distribution analysis and fracture surface observations.

## 2. Materials and Methods

The material used in this study was AA7075-T651 alloy in the form of a 5-mm-thick sheet with the chemical composition given in Table 1.

The mechanical properties of the base material were established in the tensile test on samples cut perpendicular to the rolling direction. Three samples were tested, and the obtained average values of material parameters, together with their standard deviations, are given in Table 2.

**Figure 1:**
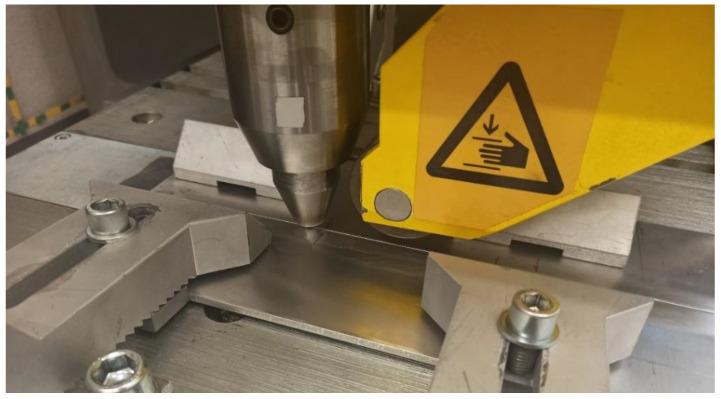
Welding system used in this study.

The material was cut into pieces with dimensions of 80 × 500 mm, with their edges precisely machined by milling. Directly before the welding process, the edges and the top surface of each sheet were degreased using benzine. The welding process was conducted on the ESAB FSW Legio 4UT system, presented in Figure 1.

To establish the influence of welding parameters on the mechanical properties of joints, twelve sets of parameters were used with various values of tool rotation speed and welding velocity, according to Table 3.

In each case, the welding direction was perpendicular to the rolling direction. The pin penetration depth was 4.8 mm. The welding tool was MX Triflute, dedicated to 5-mm-thick workpieces in butt joint configuration. The proposed set of welding parameters was based on our experience with the friction stir welding of high-strength aluminum alloys. In terms of tool rotation speed, applying a value of 1000 rpm can easily lead to excessive material flow and the formation of serious imperfections in a joint, which has been described in previous research on AA2519-T62 FSW joints [11]. At the same time, a value of rotation below 400 rpm can exert a negative effect in the form of significant softening of the stir zone, which results in overall poor performance in terms of mechanical properties. A value of welding velocity of approximately 50–100 mm/min gives stable material flow, low values of residual stresses and better clamping of welded elements during the process due to material thermal expansion ahead of the rotating tool [7,11]. The used MX Triflute tool consists of a tapered probe body and three equally spaced helical flutes [28]. The dimensions of the tool are given in Table 4.

**Figure 2:**
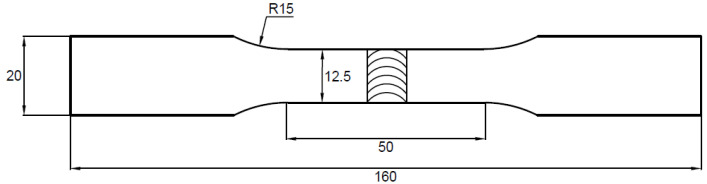
Geometry of the tensile sample.

After the welding process, samples for tensile testing and microstructure investigation were cut from the joint section. The tensile sample geometry is presented in Figure 2.

The tensile tests were carried out in accordance with the ASTM E8/E8M standard on an Instron 8802 MTL supported by WaveMatrix software (Instron, Norwood, MA, USA). The fractured surfaces of selected samples were investigated using a scanning electron microscope (SEM), namely the Jeol JSM-6610 (Jeol, Tokyo, Japan). As part of the microstructural analysis, metallographic specimens were mounted in resin and subjected to standard metallographic preparation, including grinding and polishing. Selected samples were used to establish microhardness distributions on a microhardness tester, namely the Struers DURA SCAN 70 (Struers, Copenhagen, Denmark), applying a 0.98 N load. The microhardness testing was conducted in accordance with the ASTM E384 standard. In order to reveal the microstructure, Keller’s reagent (20 mL H_2_O, 5 mL 63% HNO_3_, 1 mL 40% HF, and one drop of 36% HCl) was used, with a 15 s etching time.

## 3. Results and Discussion

### 3.1. Initial Observations

**Figure 3:**
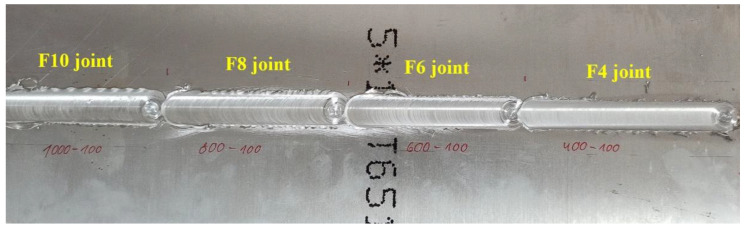
Example of welded joints obtained with 100 mm/min welding velocity.

All used sets of welding parameters allowed us to obtain defect-free weld faces. The most visible difference in the joints’ appearance was the amount of flash, which increased together with the tool rotation speed. It is impossible to state unequivocally that the welding velocity in the used range (50–100 mm/min) has any impact on the formation of flash. An example photo of the joints produced with the highest used welding velocity is presented in Figure 3.

This example illustrates the overall tendency of flash formation. For all tested values of welding velocity, the greatest flashes were obtained in joints produced with a tool rotation speed of 800 and 1000 rpm, pointing to the excessive material flow [7,11,29].

### 3.2. Mechanical Properties

**Figure 4:**
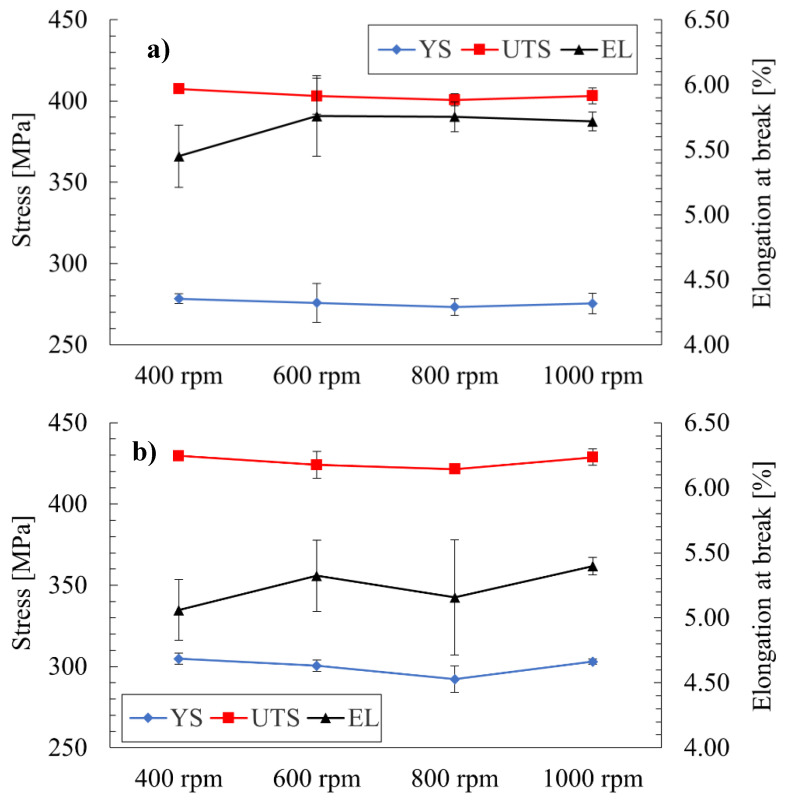

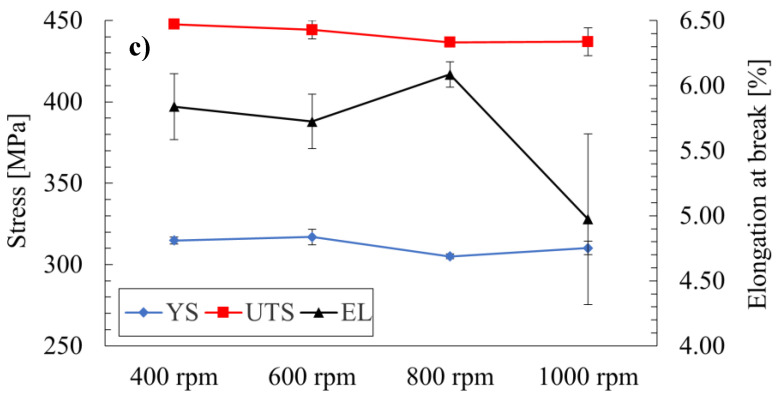
Influence of the tool rotation speed on yield strength (YS), ultimate tensile strength (UTS) and elongation at break (EL) for various values of welding speed: 50 mm/min (**a**), 75 mm/min (**b**) and 100 mm/min (**c**).

As the primary objective of this study was to establish the basic mechanical properties of joints produced using the proposed set of welding parameters, firstly, the results of the performed tensile tests were subjected to statistical treatment and are presented in the plots in Figure 4a–c.

Analyzing the obtained values of ultimate tensile strength, it can be seen that, in all cases, the values exceeded 400 MPa, corresponding to 68.5% minimum joint efficiency. The highest average value was reported for the F4 joint, equaling 447.7 ± 1.2 MPa (76.7% joint efficiency), and the lowest for the S8—400.6 ± 3.8 MPa (68.6% joint efficiency). The overall tendency can be linked to the amount of heat generated in the welding process [7]. Generally, for defect-free joints of precipitation-hardened aluminum alloys, there is a relationship between the UTS and the amount of heat generated during the welding process, defined as the heat input [7,24]. The heat input is proportional to the value of the tool rotation speed and is inversely proportional to the welding velocity. For this reason, the F4 joint, produced with the lowest value of tool rotation speed (400 rpm) and the highest value of welding velocity (100 mm/min), is characterized by the lowest heat input, which generally entails the highest UTS. Comparing the obtained results to those available in the literature, some conclusions can be drawn. Generally, the produced joints are characterized by relatively high joint efficiencies, taking into account the comparatively low values of welding velocity (up to 100 mm/min) and the material’s large thickness (5 mm). Dimopoulos et al. obtained the highest efficiency for 2-mm-thick AA7075 (around 60%) using a 1500 rpm tool rotation speed, a 110 mm/min welding velocity and a flat cylindrical pin with a 4 mm diameter [16]. For this type of welding velocity, a lower tool rotation speed (400–600 rpm) seems more appropriate in terms of heat generation, and an additional factor is that, in the current investigation, the Triflute tool was used, which generally gives better performance in joints [7,19,28]. Moreover, comparing the obtained results to data for SSFSW, it can be noticed that the parameters proposed by Li for 5-mm-thick AA7075 (1500 rpm and 30 mm/min) gave less than 70% joint efficiency [22]. The SSFSW allows us to significantly limit the amount of heat affecting the welded material, but, in this case, a better result for the considered thickness of AA7075 can be achieved by conventional FSW (Figure 4c) [8]. In terms of the UTS and elongation at break, the results in the current study are slightly lower than the properties reported by Mahoney et al. for 6.35-mm-thick AA7075 using a welding parameter of 127 mm/min and unknown values of tool rotation speed and tool type [15]. As the best results have been established in the case of the joint produced under 400 rpm and 100 mm/min, it is highly probable that the joint efficiency can be further increased by applying a higher value of welding velocity (Figure 4c) [7,8]. The reported values of the YS show that they strongly depend on the welding velocity and that their reduction is even higher than the UTS (Figure 4a–c). The average decrease in the YS has been established as 49.6, 45.2 and 43% for the tool welding speeds of 50, 75 and 100 mm/min, respectively. This reduction is the direct effect of the precipitates overaging, and similar values are reported in the literature [8,15]. An unusual aspect of the results is the average values of the mechanical properties of joints obtained via 800 rpm, which, in some cases, are lower than for 1000 rpm. Although this seems to represent an inconsistency in the effect of the tool rotation speed on the UTS [30,31], nevertheless, when the error bars are considered, such a conclusion cannot be verified (Figure 4a–c). Similar reports indicating fluctuations in the UTS average values in terms of tool rotation speed are presented in the literature [32].

**Figure 5:**
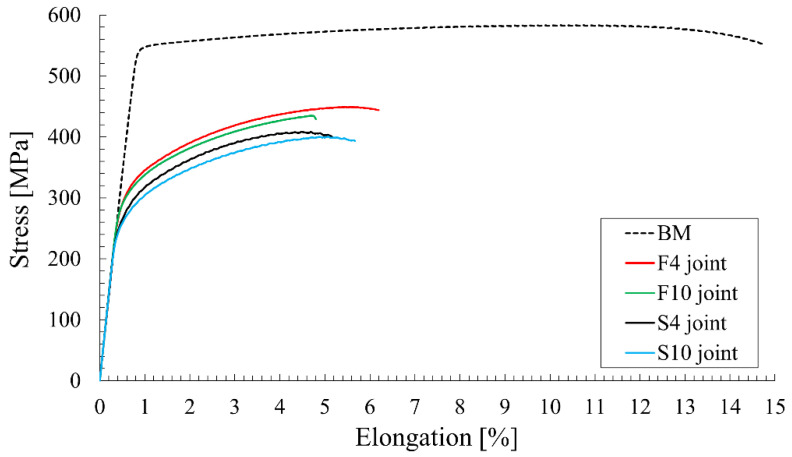
Comparison of tensile curves for the base material and joints produced with edged parameters.

The tensile curves for the base material and selected joints are presented in Figure 5.

Analyzing the obtained curves, it can be stated that increasing the welding velocity has a greater impact on the strength of the joint than a decrease in tool rotation speed (Figure 5). The produced joints are characterized by significantly lower elongation at break, but this is a result of severe strain localization in the weakest zone, commonly occurring in welded joints [18]. The weakest zone of the joints is described in the following sections of the paper.

### 3.3. Macro- and Microstructure Analysis

**Figure 6:**
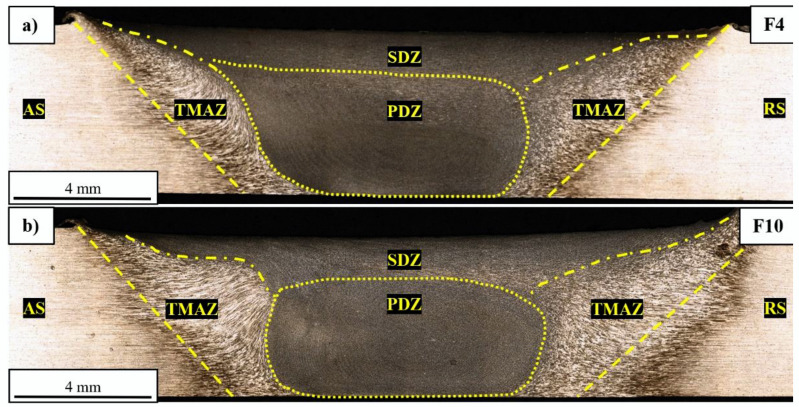

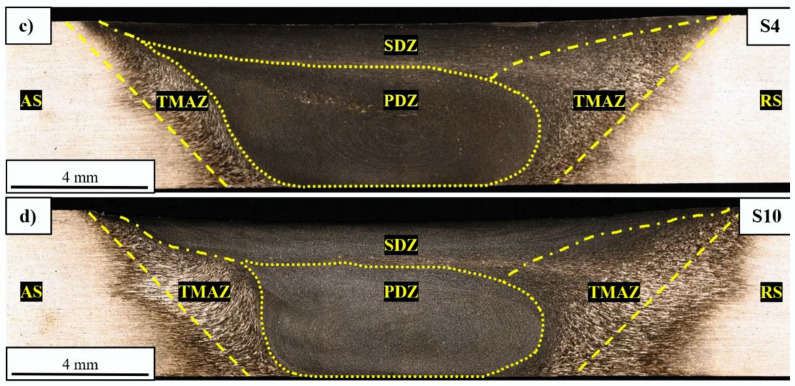
Macrostructural images of joints F4 (**a**), F10 (**b**), S4 (**c**) and S10 (**d**), together with estimated localizations of the SDZ, PDZ and TMAZ. Retreating (RS) and advancing side (AS) are marked on the images.

In order to identify the impact of the FSW process on the grainy structure of the welded alloy, macro- and microstructural analysis were performed. The macrostructural images of the selected (edged) samples are presented in Figure 6a–d.

Analyzing the obtained images, it can be seen that there are no imperfections and the joints’ structures are typical of the FSW process (Figure 6a–d). Based on the relation between the rotation and welding directions, we can distinguish the advancing side (AS), where the linear velocity vector of the rotating tool and the welding direction are the same, and the retreating side (RS), where the linear velocity vector of the rotating tool and the welding direction are opposite to each other [7]. Some differences between the two sides can be observed, mostly in terms of the stir zone (SZ) shape (Figure 6a,c). As is typical for the FSW process, the SZ consists of fine, recrystallized grains, which were considered in the microstructural analysis. Depending on the origin of the SZ, two main subareas could be distinguished, a shoulder-driven zone (SDZ) and a pin-driven zone (PDZ), where, in the first case, the grain refinement occurred as the result of shoulder affection and, in the second case, via stirring pins [33]. The participation of these zones differs for various sets of used parameters, although the exact proportion is difficult to estimate. It can be seen that the size of the SZ increases with the decrease in tool rotation speed (Figure 6a,b) and welding velocity (Figure 6a,c). The size of each zone was measured at the mid-thickness (2.5 mm) of the joints and the results are presented in Table 5.

The obtained results show an overall tendency to decrease the size of the SZ along with increasing tool rotation speed, mostly visible in the case of joints produced with the lowest value of welding velocity. This decrease comes at the expense of increasing the TMAZ size (Table 5). It can be noticed that an increase in the tool rotation speed from 400 to 1000 rpm gives a 25% increase in the TMAZ width at the AS. The grainy structure of the TMAZ illustrates the material flow during the stirring process, and it is further discussed in the next section of the paper.

**Figure 7:**
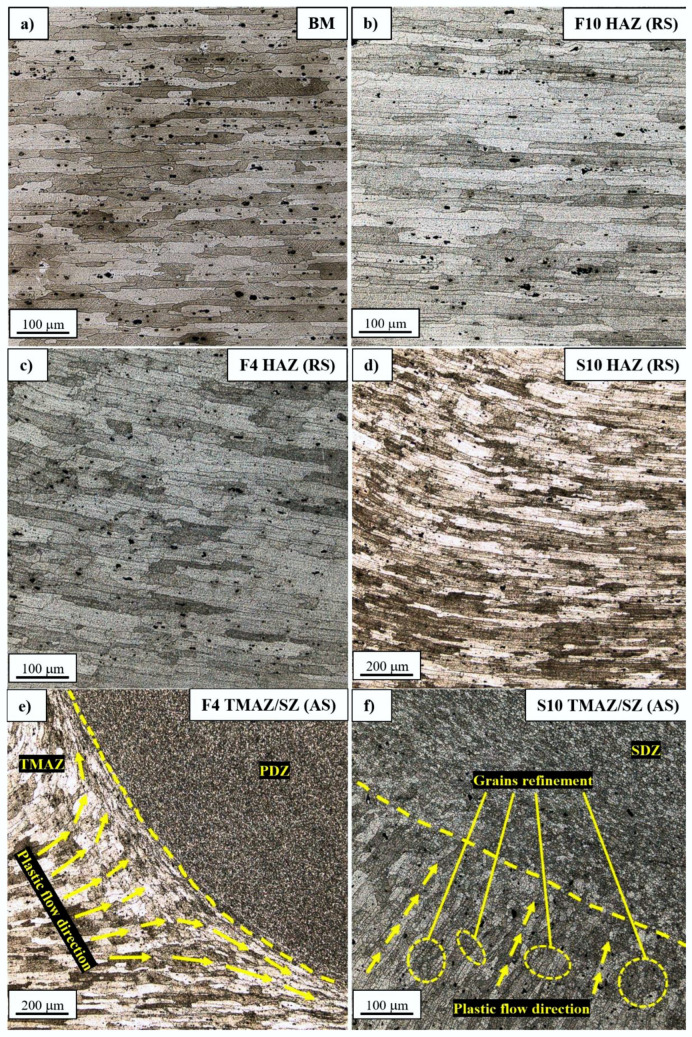

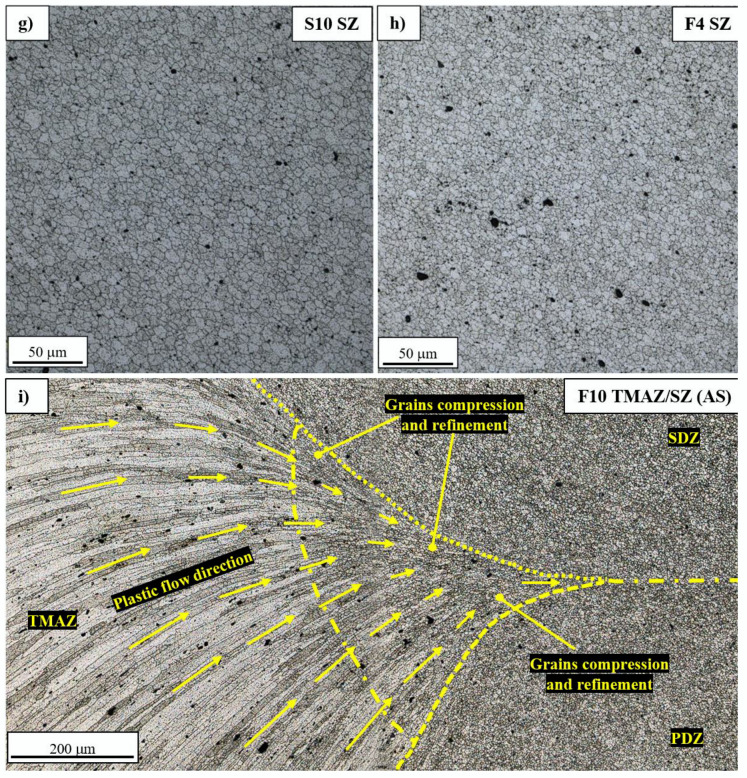
Microstructural images of the base material (**a**), HAZ in the F10 (**b**), F4 (**c**) and S10 (**d**) joints, TMAZ/SZ interface in the F4 (**e**) and S10 joints (**f**), SZ in the F4 (**g**) and S10 joints (**h**) and TMAZ/SZ interface in the F10 joint (**i**).

Selected microstructural images are presented in Figure 7a–e.

The base material’s microstructure is characterized by elongated grains with a size of approximately 73.2 ± 39.9 μm (Figure 7a). Analyzing the HAZ in the F10 sample, closest to the TMAZ, it can be stated that the grain size is approximately 113.5 ± 58.7 μm, so there is more than a 50% increase in the average value (Figure 7b). Although the most important phenomenon, which takes place in the HAZ of precipitation-hardened aluminum alloys joined via FSW, is the overaging of the strengthening phase, the evolution of grainy structures also has an impact on the joints’ properties [8,15]. Mostly, it concerns the fatigue properties, because larger grains make it easier for a crack to propagate [34]. It also influences the overall strength in the affected area, which it is presented in the microhardness analysis, later in the paper. The decrease in the tool rotation speed to the value of 400 rpm results in 79.6 ± 28.5 μm grain size (Figure 7c), which is only slightly lower than the base material’s value. At the same time, the decrease in the welding velocity to 50 mm/min strongly promotes the grain growth in the HAZ, giving a grain size of 114.9 ± 43.56 μm (Figure 7d). It should be noted that samples F10 and S10 are characterized by a very similar grain size in the HAZ, despite the differences in the used value of tool rotation speed. In the investigated set of welding parameters, the welding velocity has a stronger effect on the HAZ’s grainy structure.

The analysis of the boundary between the TMAZ and SZ at the bottom part of the F4 sample allows us to observe the plastic flow of the welded material (Figure 7e) [35]. In this area, the grains have been severely deformed, creating a layer of compressed grains surrounding the PDZ with a thickness of approximately 100 μm. At the same time, in the S10 sample, the highly deformed grains in the TMAZ undergo partial dynamic recrystallization (Figure 7f), which is promoted by higher heat input.

The SZ of the S10 sample has, typical for the FSW process, a microstructure with equiaxed, ultrafine grains with a size of 6.3 ± 2.9 μm (Figure 7g). A similar grain size has been reported by many authors investigating 7075 joined via FSW, including Li (8 μm) [22], Iwaszko (7.6 ± 1 μm) [20], Feng (6.7–4.6 μm, depending on parameters) [26], Yeni (4–6 μm) [17] and Sun (2 μm) [23]. It should be noted that the S10 sample is characterized by the highest heat input from all used sets of welding parameters. In this study, we did not aim to establish a relationship between the process parameters and the SZ’s grain size; nevertheless, the measurements for the F4 sample (lowest heat input) gave the result of 5.2 ± 1.7 μm (Figure 7h). This suggests the tendency to slightly decrease the SZ grain size along with decreasing heat input in the used set of welding parameters. It confirms the literature data, and similar observations have been made by Feng [26]. The grainy structure that attracts attention is presented in Figure 7e. In the upper part of the F10 sample’s TMAZ/SZ, the plastic flow dragged grains into the area between the SDZ and PDZ, where they were severely compressed and underwent partial dynamic recrystallization. The participation of recrystallized grains changed along with the plastic flow, and, at the end, ultrafine grains were formed at the SDZ/PDZ interface (Figure 7e). This structure was observed only in the F10 sample. In terms of joining high-strength aluminum alloys via the MX Triflute tool, void imperfections can occur at this location, when the welding parameters are not set properly. It was observed by the authors of one of the previous studies concerning AA2519-T62 alloy that the formation of voids occurred due to discontinuities in the material flow (high values of both tool rotation and welding speed cause differences in the temperature between the top and bottom parts of the welded workpiece) [11]. It is possible that a further increase in welding velocity would strongly affect the upper part of the TMAZ/SZ interface and imperfections are likely to be formed, but additional experiments are required to confirm this.

For microhardness and fractography analysis, two samples were selected: F4 (lowest heat input, high joint efficiency) and S10 (highest heat input, low joint efficiency).

### 3.4. Microhardness Distribution

**Figure 8:**
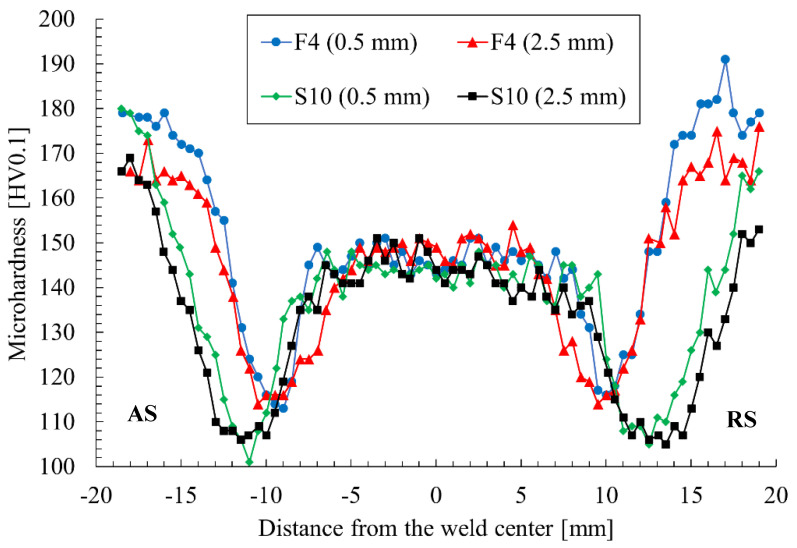
Microhardness profile of the FSW joints at the distance of 0.5 and 2.5 mm from the weld face.

The microhardness profiles of the F4 and S10 samples are presented in Figure 8.

The microhardness of the base material has been established as 170 ± 3.6 HV0.1. As can be observed in the microhardness profile, the FSW process reduced the base material’s microhardness in the entire joint. The microhardness of the SZ was within the range of 140–150 HV0.1 for both samples, although the F4 joint seemed to have a slightly higher value, which could be an effect of the finer grains, as described in the previous section. The highest reduction in microhardness occurred at the boundary between the HAZ and TMAZ, which can be identified as the low-hardness zone (LHZ). Depending on the parameters of the FSW process, it can be located at the HAZ/TMAZ interface, in the TMAZ or in the SZ [36,37]. The LHZ is often the location where the tensile failure of FSW joints occurs [38]. The lowest reported values of microhardness are approximately 100–105 HV0.1 for the sample obtained with the highest heat input and are located from 10 to 15 mm from the joint center. In the case of the F4 sample, the reduction in microhardness is slightly lower and the reported value is around 115 HV0.1, and it is also located closer to the center of the weld, at a distance of 10 mm. A similar decrease in microhardness has been reported by Iwaszko (approximately 105 HV0.1) [20], and, also in terms of the SSFSW, Li obtained almost identical values but at the closest distance to the weld center (5 mm) [22]. Applying high-speed FSW allowed Zhang to significantly increase the microhardness of the LHZ, to above 140 HV0.1 [21]. It also can be observed that the HAZ of the S10 sample in the present study extends beyond the investigated area. An additional conclusion that can be drawn is that, at various distances from the weld face, there are slight changes in the microhardness. Generally, the material closer to the tool’s shoulder exhibits greater losses in microhardness (Figure 8). The most important conclusion is that the microhardness and location of the LHZ strongly depend on the used set of welding parameters. The crucial factor is the heat input of the welding process, as it causes the overaging of the strengthening phase and grain growth. As was mentioned in the discussion of the mechanical properties, a lower tool rotation speed and higher welding velocity decrease the amount of heat generated during the process [7,24]. In the investigated sets of parameters, the best results in terms of microhardness overlap with the highest UTS and correspond to the weld produced with a 400 rpm tool rotation speed and 100 mm/min welding velocity (Figure 4c and Figure 8).

### 3.5. Fractography of the Tensile Samples

**Figure 9:**
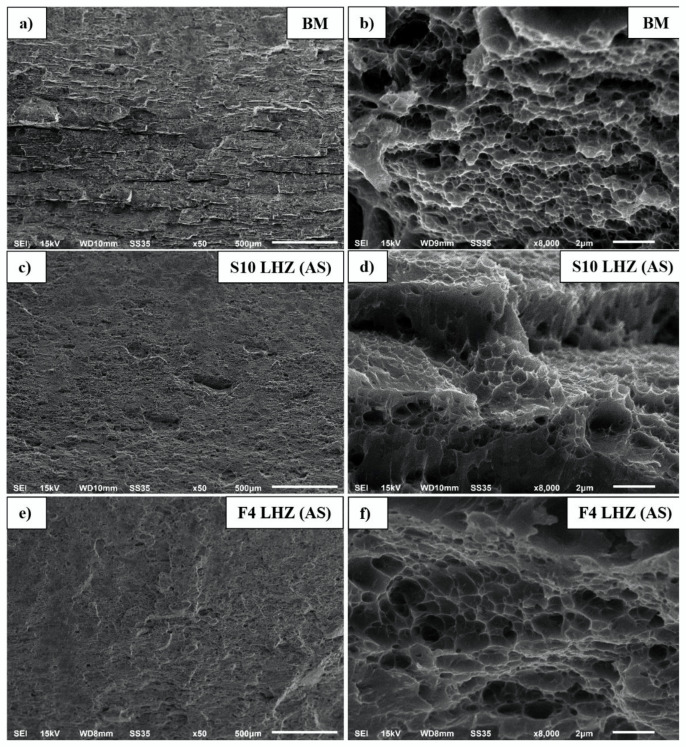
Fracture surfaces of tensile samples: BM (**a**,**b**), S10 (**c**,**d**) and F4 (**e**,**f**).

The fractography analysis involved two samples, previously discussed in the microhardness distribution section: S10 and F4. The selected areas of the fracture surfaces are presented below in Figure 9a–f.

The fracture surface of the base material (Figure 9a) is characterized by long, flat regions, which correspond to the elongated grains (Figure 7a), separated by ductile tear ridges. Although the fracture has some participation of intergranular cracking (Figure 9a), the overall mechanism is the microvoid coalescence (MCV), giving a typical fine-dimpled structure (Figure 9b) [39]. All tensile samples tested in this investigation failed at the TMAZ/HAZ interface at the AS, which corresponds to the LHZ identified in the previous section (Figure 8). Compared to the base material, the tested joints have generally smooth fracture surfaces (Figure 9c,e). In both samples, the fracture mechanism is dominated by a transgranular ductile rupture, which is reflected in the mixed participation of large and fine dimples (Figure 9d,f) [40]. The locations of failure in these two samples are the same and the only difference is their microhardness: 100–105 HV0.1 in the case of S10 and 115 HV0.1 for F4. Besides the difference in fracture mechanism between the base material and the LHZ, no direct distinctions in the fracture surface of the S10 and F4 samples can be clearly determined (Figure 9d,f); nevertheless, the differences in microhardness promote the more ductile fracture of the S10 sample.

## 4. Conclusions

The following conclusions can be drawn from this study:

1. All obtained joints were defect-free. In all cases, the values of UTS exceeded 400 MPa, corresponding to 68.5% minimum joint efficiency. For this reason, the used sets of welding parameters (welding velocities: 50, 75 and 100 mm/min and tool rotation speeds: 400, 600, 800 and 1000 rpm) are suitable for producing good-quality welds of 5-mm-thick AA7075-T651 in butt joint configuration.
2. The highest value of UTS—447.7 MPa (76.7% joint efficiency)—has been reported for the joint produced with a 400 rpm tool rotation speed and 100 mm/min welding velocity.
3. The used parameters influence the microstructure of the produced joints. For the strongest obtained joint, the SZ has a 5.2 ± 1.7 μm grain size and the LHZ (115 HV 0.1) is located on the TMAZ/HAZ interface at the distance of 10 mm from the center of the weld.
4. All tensile samples tested in this investigation failed in the LHZ at the AS. The fracture mechanism is dominated by the transgranular ductile rupture with microvoid coalescence.

## Figures and Tables

**Table 1:** Chemical composition (wt.%) of AA7075-T651 alloy.

Si	Fe	Cu	Mn	Mg	Cr	Zn	Ti	Al
0.071	0.122	1.610	0.025	2.596	0.197	5.689	0.041	Base

**Table 2:** Basic mechanical properties of AA7075-T651 alloy.

Yield Strength, R_0.2_	Tensile Strength, R_m_	Elongation, A
547.5 ± 1.3 MPa	583.5 ± 1 MPa	14.4 ± 0.6%

**Table 3:** Welding parameters and sample designations.

Welding Velocity	Tool Rotation Speed			
	400 rpm	600 rpm	800 rpm	1000 rpm
**50 mm/min** (*slow*)	S4	S6	S8	S10
**75 mm/min** (*medium*)	M4	M6	M8	M10
**100 mm/min** (*fast*)	F4	F6	F8	F10

**Table 4:** The dimensions of the used tool.

**Shoulder Profile**	Spiral
**Shoulder Diameter**	19 mm
**Pin Profile**	Threaded and tapered with three spiral flutes
**Pin Length**	4.8 mm
**Pin Diameter**	6.5–8.7 mm

**Table 5:** The sizes of welded zones at mid-thickness of different joints.

Joint	Measured Zone		
	SZ	TMAZ (AS)	TMAZ (RS)
**F4**	8.1 mm	2.4 mm	3.4 mm
**F10**	8 mm	3 mm	3.3 mm
**S4**	9 mm	2 mm	3 mm
**S10**	7.9 mm	2.55 mm	3.6 mm

## Data Availability

Not applicable.
